# Postnatal Serum Total Thyroxine of Very Preterm Infants and Long-Term Neurodevelopmental Outcome

**DOI:** 10.3390/nu13041055

**Published:** 2021-03-24

**Authors:** Yung-Chieh Lin, Chen-Yueh Wang, Yu-Wen Pan, Yen-Ju Chen, Wen-Hao Yu, Yen-Yin Chou, Chi-Hsien Huang, Wei-Ying Chu, Chyi-Her Lin, Osuke Iwata

**Affiliations:** 1 Department of Pediatrics, National Cheng Kung University Hospital, College of Medicine, National Cheng-Kung University, Tainan 70457, Taiwan; drapple@mail.ncku.edu.tw (Y.-C.L.); panyuwen0527@hotmail.com (Y.-W.P.); yensweet@gmail.com (Y.-J.C.); fieldof19@gmail.com (W.-H.Y.); yenyin@mail.ncku.edu.tw (Y.-Y.C.); 2 Department of Pediatrics, College of Medicine, National Cheng-Kung University, Tainan 70457, Taiwan; 3 Department of Pediatrics, Tainan Sinlau Hospital, Tainan 70142, Taiwan; peddrwang@gmail.com; 4 Department of Family Medicine, E-Da Hospital, Kaohsiung 82445, Taiwan; evaairgigaa@gmail.com; 5 School of Medicine for International Students, College of Medicine, I-Shou University, Kaohsiung 82445, Taiwan; 6 Department of Pediatrics, Tainan Hospital, Ministry of Health and Welfare, Tainan 70043, Taiwan; raychu9629@gmail.com; 7 Department of Pediatrics, E-Da Hospital, Kaohsiung 82445, Taiwan; 8 Center for Human Development and Family Science, Department of Neonatology and Pediatrics, Nagoya City University Graduate School of Medical Science, Nagoya, Aichi 467-8601, Japan

**Keywords:** very preterm infants, thyroid function, neurodevelopmental outcome, newborn screening, hypothyroxinemia

## Abstract

Primary congenital hypothyroidism is a disease associated with low serum thyroxine and elevated thyroid-stimulating hormone (TSH) levels. The processes of screening and treating congenital hypothyroidism, in order to prevent neurodevelopmental impairment (NDI) in newborns, have been well investigated. Unlike term infants, very preterm infants (VPIs) may experience low thyroxine with normal TSH levels (<10.0 μIU/mL) during long-stay hospitalization. In the current literature, thyroxine treatment has been evaluated only for TSH-elevated VPIs. However, the long-term impact of low thyroxine levels in certain VPIs with normal TSH levels deserves more research. Since July 2007, VPIs of this study unit received screenings at 1 month postnatal age (PNA) for serum TSH levels and total thyroxine (TT4), in addition to two national TSH screenings scheduled at 3–5 days PNA and at term equivalent age. This study aimed to establish the correlation between postnatal 1-month-old TT4 concentration and long-term NDI at 24 months corrected age among VPIs with serial normal TSH levels. VPIs born in August 2007–July 2016 were enrolled. Perinatal demography, hospitalization morbidities, and thyroid function profiles were analyzed, and we excluded those with congenital anomalies, brain injuries, elevated TSH levels, or a history of thyroxine treatments. In total, 334 VPIs were analyzed and 302 (90.4%) VPIs were followed-up. The postnatal TT4 concentration was not associated with NDI after multivariate adjustment (odd ratios 1.131, 95% confidence interval 0.969–1.32). To attribute the NDI of TSH-normal VPIs to a single postnatal TT4 concentration measurement may require more research.

## 1. Introduction

The importance of thyroid function screenings (TFS) in newborns has been extensively researched [1,2]. Infants deprived of appropriate treatment for elevated thyroid-stimulating hormone (TSH) and low thyroxine may suffer long-term neurodevelopmental sequela. All full-term newborns need to be screened soon after birth for thyroxine (T4) or thyroid-stimulating hormones in relation to the nations’ newborn screening polices [3,4,5].

Unlike full-term infants, very preterm infants (VPIs; <31 weeks’ gestational age) may display false-negative screenings at birth due to the incomplete development of the hypothalamic–pituitary axis [6]. Two forms of thyroid function anomaly may occur in VPIs. One is delayed elevated TSH [7,8], and the other is transient hypothyroxinemia of prematurity (THOP) [9,10]. Therefore, certain very preterm infants may experience normal TSH with low serum thyroxin for weeks.

Concerning this result, which potentially affects the brain development of long-hospitalized very preterm infants, several thyroxine-replacement studies have been conducted. However, the results are inconclusive in terms of improving the long-term neurodevelopmental outcome [11,12,13,14,15]. According to the current consensus [8,16], very preterm infants with elevated TSH (≥10.0 μIU/mL) are recommended to undergo a further follow-up regardless of thyroxine concentration, and should be considered for treatment.

In brief, most scholars seem to agree on the need for an extra screening during admission [10]. However, in those VPIs with TSH < 10.0 μIU/mL, few studies have investigated the level of thyroxin that necessitates thyroxin supplementation. Williams et al. voiced concerns regarding the current clinical screening protocols [17]; however, relatively little is known about the optimal timing of extra postnatal screening beyond national newborn screening policies. Furthermore, the effect of any renewal protocol on long-term neurodevelopmental outcomes should be further investigated along with the neonatal morbidities of prematurity [7,9].

In Taiwan, all newborns receive TSH screening after birth, and preterm infants are requested to attend a second TSH screening at term equivalent age [3]. In addition to the two national screenings, the unit of this study developed a TFS protocol at 1 month postnatal age (PNA) for those long-stay VPIs for whom the national screenings might have missed the diagnosis of delayed elevated TSH (detailed in the methodology, Section 2.2). The developed screening protocol involves serum total thyroxine (TT4) and TSH. The additional protocol provides an important opportunity to reassess the effect of postnatal TT4 on the long-term neurodevelopmental outcome of very preterm infants. As such, this study aims to explore the link between TT4 concentration at 1 month and the neurodevelopmental outcome at 24 months corrected age among very preterm infants with serial normal TSH and normal cranial ultrasound results.

## 2. Materials and Methods

### 2.1. Study Design and Eligibility Criteria

The study aimed to identify the relationship between TT4 concentration and long-term outcomes for VPIs, born at a gestational age (GA) equal to or less than 30 weeks. The data of VPIs were retrospectively collected by reviewing medical records in January 2019, when the participants had all completed the routine 24-month corrected age follow-up. The study analysis was undertaken in April 2019. The exclusion criteria are listed below:

- Age at admission greater than 7 days old;
- Death at discharge;
- Severe brain injury, such as severe intraventricular hemorrhage (IVH) or periventricular leukomalacia (PVL), confirmed by serial cranial ultrasounds or magnetic resonance imaging, which was related to the poor neurodevelopmental outcome;
- Congenital anomaly or syndromic gene anomaly;
- Any event of elevated TSH (≥10 μIU/mL) at serum or blood spot test;
- Infants treated with L-thyroxine during admission;
- T4 and TSH data not simultaneously available during admission.

### 2.2. Study Setting and Thyroid Function Survey for VPIs

This study unit was a 20-bed tertiary neonatal intensive care unit (NICU) at the National Cheng Kung University Hospital in Tainan, Taiwan. The annual care volume of this unit is approximately 350–400 neonatal admissions, including approximately 60 VPIs. Two neonatologists, two residents, and one nurse practitioner are regularly in charge of the admitted infants each month [18].

The thyroid functions of VPIs in this study were screened in three stages, as follows.

- Stage I: all admitted newborns receive first national thyroid screening at PNA 3–5 days, along with a TSH concentration screening. TSH concentration is measured by heel blood sampling filter paper cards via a tandem mass spectrometer in the national laboratory [3].
- Stage II: for the survey of delayed TSH elevation in VPIs, the VPIs in this study unit undergo a TFS at 1 month PNA, including assessment for serum total T4 (TT4) and TSH concentration, which were measured by radioimmunoassay. This protocol has been in place since July 2007. Therefore, the VPIs that were evaluated for enrollment were born between August 2007 and July 2016.
- Stage III: preterm infants are forced to take a second national screening scheduled for their term equivalent age (TEA, GA 37–42 weeks), also assessing body weight (≥2200 g), regardless of the first TSH screening’s results [3].

### 2.3. Clinical Variables Collection

#### 2.3.1. Maternal and Antenatal Variables

The maternal medical history included maternal age, maternal education level, pregnancy complications, medication history, maternal gestational diabetes mellitus (GDM), and maternal pre-eclampsia.

#### 2.3.2. Variables during Perinatal Period

The perinatal data included GA, bodyweight at birth, sex, z score of birth bodyweight, Apgar score at 1 and 5 min after birth, method of delivery, and vital signs in the first few hours of birth. Anthropometry z scores were based on reference data from the website [19,20].

#### 2.3.3. Morbidity Variables during Hospitalization

Data on respiratory distress syndrome (RDS), patent ductus arteriosus (PDA), sepsis, intraventricular hemorrhage (IVH), necrotizing enterocolitis (NEC) [21], retinopathy of prematurity (ROP), postnatal steroids therapy, chronic lung disease (CLD) [22], periventricular leukomalacia (PVL), duration of invasive ventilation, and postnatal steroid therapy for CLD, respiratory support status, PNA and postmenstrual age (PMA) were obtained at discharge [18,23].

#### 2.3.4. Thyroid Function Data

PNA and PMA at the screening, serum TT4 concentration, serum TSH concentration, and the results of the two national TSH screenings were recorded.

### 2.4. Primary Outcome

Infants were assessed by a single team at 24 months corrected age using the Bayley Scales of Infant Development, Second Edition (BSID-II), or the Third Edition (BSID-III), as in the previous reports [24,25,26,27]. The Bayley-II scales were used for VPIs born in 2007–2010, while the Bayley-III scales were for VPIs from 2011–2016. Neurodevelopmental impairment (NDI) was defined by any one of the criteria below:

- Any form of cerebral palsy;
- Mental or motor development indices < 70 in the Bayley-II scales;
- Any domain of composite score < 85 in the Bayley-III scales.

The cognition and language composite scores of the BSID-III were combined into a predicted mental developmental index (MDI), according to the algorithm suggested by Moore et al. [26]. The predicted MDI synthesized from the BSID-III cognition and language scores, along with the MDI in the BSID-II, were used to represent the mental performance of the VPIs [24]. The dependence of MDI on TT4 concentration was analyzed with linear regression analysis.

### 2.5. Statistical Analysis

All analyses were conducted using SPSS (v.26, IBM, Armonk, NY, USA). The dependence of neurodevelopmental impairment on the clinical variable and TT4 was first assessed by univariate analysis, after adjusting for a priori variables. Covariates were chosen for multivariate analysis after taking into account the clinical relevance and collinearity between the chosen variables. Multivariate logistic regression analyses were performed to evaluate the crude effects of the potential independent variables on the NDI. A *p*-value of less than 0.05 was considered significant.

## 3. Results

### 3.1. The Enrollment of Neurologically Intact Infants with Available Thyroxine Data during Hospitalization and Three Consecutively Normal TSH Screenings

**Figure 1:**
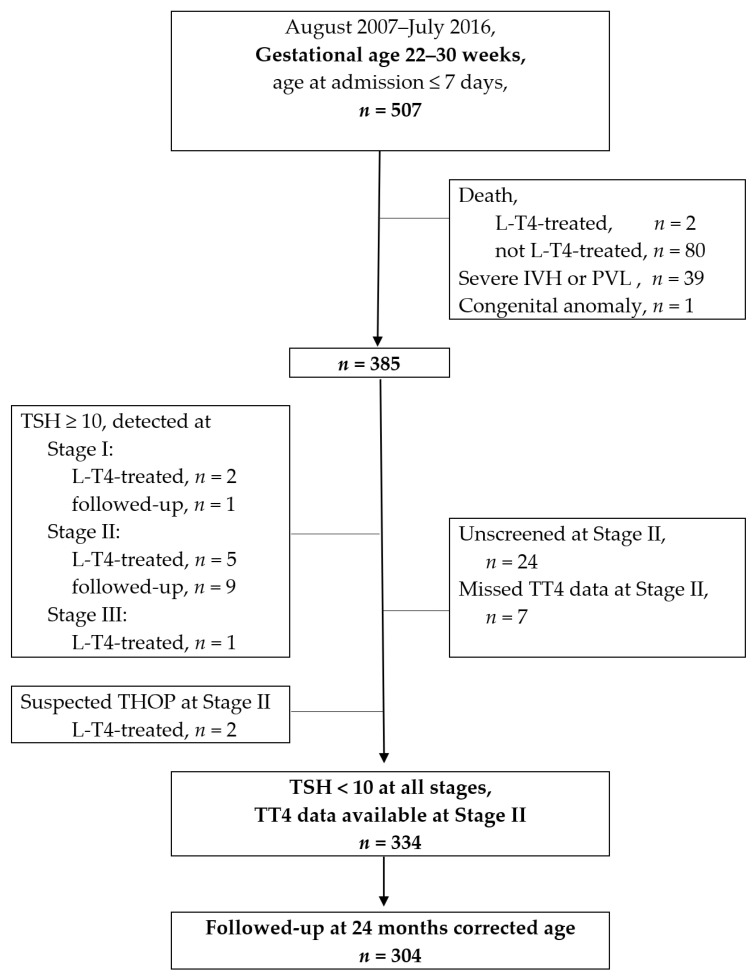
Flow chart of patient enrollments. L-T4: levothyroxine; IVH: intraventricular hemorrhage; PVL: periventricular leukomalacia; TSH: thyroid-stimulating hormone (unit: μIU/mL); TT4: total serum thyroxin; THOP: transient hypothyroidism of prematurity; a detailed description of the stages presented in the methodology, Section 2.2.

In the 9-year study period, 507 very preterm infants born at 22–30 weeks gestation were admitted to the study unit (shown in Figure 1). The median age of mortality was 3.5 days, and there was an 87% of mortality within the PNA 1 month, which was earlier before the time of Stage II. Two of the 82 mortal patients ever received any thyroxine supplement.

A total of 385 VPIs were further reviewed for their thyroid function profiles after eliminating cases of death, severe IVH and congenital anomaly. At Stage I, three VPIs had a TSH ≥ 10 μIU/mL; one passed after a rescreening, and two received thyroxine supplements. At Stage II, 14 VPIs had a TSH ≥ 10 μIU/mL; 9 passed after a rescreening, and 5 received thyroxine supplements. At Stage III, one VPI had TSH ≥ 10 μIU/mL and received thyroxine supplements. Two VPIs were assessed as having suspected THOP at Stage II, and were treated according to the endocrinologist’s suggestion. Supplementary Table S1 shows the 12 VPIs who received any thyroxine supplementation in this study. None of the survivors had developed permanent congenital hypothyroidism by the time of the 2–3 years follow-up. An extended and detailed discussion of the thyroxine-treated babies is beyond the scope of this paper.

This study excluded 24 infants who were unscreened at Stage II and 7 infants without TT4 data at Stage II. A total of 334 participants met the eligibility criteria of having neurologically intact brains, TT4 data, and consecutive normal TSH levels (<10 μIU/mL). The 334 VPIs were analyzed to test the correlation between postnatal TT4 and neurodevelopmental impairment. In total, 302 of 334 (90.4%) VPIs received an evaluation in the follow-up clinic at 24 months corrected age.

### 3.2. The Clinical Characteristics of Enrolled Very Preterm Infants

Table 1 shows the clinical characteristics of the 334 infants in the study cohort. Of these, 91 neonates (27.1%) developed NDI, including seven infants with any form of cerebral palsy (CP) and 84 infants with neurodevelopmental delay and without CP. The average postnatal age of infants that received thyroid screening under the implemented protocol was 30.3 ± 5.5 days. Supplementary Table S2 shows that the z scores of birth bodyweight and postmenstrual age of sampling were significantly positively correlated with TT4 concentration, after adjustment with multivariate analyses. Compared to female VPIs, male infants had statistically significantly lower TT4 concentrations.

### 3.3. The Correlation between Neurodevelopmental Impairment and the TT4

Table 2 depicts the univariate logistic regression analysis of the 334 infants, undertaken to investigate the association between clinical variables and neurodevelopmental impairment. The variables, which included antenatal antihypertensive drugs, maternal pre-eclampsia, surfactant-treated respiratory distress syndrome, history of treatment for hsPDA, necrotizing enterocolitis ≥ stage 2, history of treatment for retinopathy of prematurity, chronic lung disease, days on invasive ventilation, and postnatal steroid use for chronic lung, were significantly associated with neurodevelopmental impairment (all *p* < 0.05, respectively). Infants who had received antenatal steroids, and had a higher maternal school level, GA, birth bodyweight, and z score of bodyweight at discharge, were less likely to be associated with neurodevelopmental impairment (all *p* < 0.05, respectively).

As shown in Table 3, after using the multivariate model, the incidence of NDI at 24 months corrected age was shown to significantly correlate with various clinical variables, including male infants (odds ratio (OR) 1.889; 95% confidence interval (CI) 1.067–3.343, *p* = 0.029), history of treatment for hsPDA (OR 1.883; 95% CI 1.002–3.54; *p* = 0.049), postnatal steroid therapy for CLD (OR 3.206; 95% CI 1.185–8.671, *p* = 0.028), and necrotizing enterocolitis (NEC) ≥ stage 2 (OR 3.839; 95% CI 1.142–12.906; *p* = 0.030). Infants with a higher maternal educational level had a lower OR of NDI (OR = 0.407; 95% CI 0.235–0.705; *p* = 0.001) after adjustment for multiple comparisons. Contrary to our expectations, the 1-month-old thyroxine concentration did not correlate with NDI in this multivariate model.

Supplementary Table S3 shows that maternal education level was positively correlated with mental performance scores (mean coefficient = 8.513, *p* < 0.001), whereas male and NEC ≥ stage 2 had a negative correlation with mental performance scores (mean coefficient = −4.970, *p* = 0.002; mean coefficient = −7.984, *p* = 0.041; respectively). TT4 concentration did not significantly correlate with mental performance scores (*p* = 0.356).

## 4. Discussion

In this retrospective study, we have reported a lack of association between a single postnatal 1-month-old TT4 concentration measurement and neurodevelopmental impairment at 24 months corrected age, for VPIs without severe brain injuries. Long-term neurodevelopmental impairment was associated with the morbidities of prematurity and being a male VPI. In the presence of serial TSH levels < 10.0 μIU/mL, the impact of the measured TT4 concentration of VPIs on long-term neurodevelopmental outcomes may require further research. However, several points are worthy of note.

### 4.1. The Choice between Total Thyroxine and Free Thyroxine for Thyroid Function Surveys

For thyroid hormone screenings in very preterm infants, the choice between total thyroxine and free thyroxine (FT4) following national newborn screenings is still under debate. To the best of our knowledge, there is a lack of large-scale research that addresses whether total thyroxine [17,28] or free thyroxine [13,29,30,31] is optimum for use in postnatal screening, even if free thyroxine is a more active component than total thyroxine. For detecting delayed TSH elevation, in this study, we chose total thyroxine with TSH as the additional marker to be used between the two phases of the national screening policy. As such, determining whether TT4 or FT4 is more suitable might be beyond the scope of this observational article.

Importantly, Flores-Robles et al. suggested that preterm infants may require lower TT4 cutoff values when undergoing newborn screenings [32]. Preterm infants with consistent thyroid dysfunction should be assessed using multipoint analyses [6,17]. In brief, the authors of this study concluded that the time series of thyroxine concentration screenings, with cutoff values specific to VPIs, is crucial, regardless of whether TT4 or FT4 is chosen as the screening marker.

### 4.2. The Timing of Thyroxine Treatment in Very Preterm Infants

VPIs have been characterized by a temporary reduction in T4 that may last for 6–8 weeks, whereas their TSH remains low to normal [28]. There is a continuing debate regarding how harmful low thyroxine concentrations are to the growing brain in very preterm infants. However, several randomized trials of thyroxine supplements, which did not consider THOP status, failed to improve the long-term neurodevelopmental outcome in treatment groups [11,12,13,33]. Briet et al. further demonstrated that thyroxine supplements were associated with more developmental problems in children of 29 weeks gestation [14]. Furthermore, thyroxine replacement in preterm infants has been reported to be harmful and related to circulatory collapse [34,35].

The possible reasons for the discrepancy in results between studies of thyroxin supplements might include the definition of THOP employed, or the use of adjustment with morbidities of prematurity. To date, the recommendation for treating THOP only applies if THOP is accompanied by TSH elevation [8,16]. F. Williams et al. attempted to define a concentration of total T4 that is less than the 10th percentile of the cord total T4 of the equivalent gestational age had the infant remained in utero [36]. The authors of this study regarded this standard proposed by Williams et al. as a good therapeutic consideration.

As can be seen in Supplementary Table S2, TT4 concentration was significantly correlated with the postmenstrual age of serum sampled. As such, the authors of this study further suggested that VPIs with low FT4, or those with low TT4 and normal TSH, should receive tailor-made thyroxine supplements. The specific therapy should take PMA, sex, z score of birth bodyweight, and morbidities of prematurity into account. However, more research is needed to support this hypothesis, and screening protocols for specific gestational age should be considered.

### 4.3. Thyroxine Concentration and Long-Term Neurodevelopmental Outcome

Very preterm infants may experience a nadir duration of serum thyroxine without elevated TSH after birth [6,32]. The impact of the nadir duration on the rapidly growing brains of VPIs remains unclear, especially when solely using observational studies from term infants. In our study, TT4 concentration was not an important cause of long-term neurodevelopmental impairment. Our findings are consistent with previous research [28,37,38].

Contrary to general expectations, some studies have even reported higher postnatal thyroxine concentrations, as opposed lower concentrations, to be a marker of adverse neuropsychological development in childhood [29,39]. Chung et al. argued that thyroid dysfunction generally does not affect neurodevelopmental outcomes, with the exception of persistent hyperthyrotropinemia [33]. However, a recent study has demonstrated low free thyroxine concentrations (<10 pmol/L) in infants to be associated with poor long-term neurodevelopmental outcomes at 3 years of age [30]. Recently, Williams et al. carried out an extensive study of the role of postnatal total thyroxine (TT4) levels obtained at four time-points without a consideration of morbidities of prematurity. Preterm infants with consistent and mild thyroid dysfunction scored less on neurodevelopmental tests at 24 months of age [17]. Altogether, there are still many unanswered questions about the thyroxine concentrations of VPIs and the long-term neurodevelopmental outcomes. Future research should focus on a time serial thyroid function screening protocol in VPIs, with long-term follow-up.

### 4.4. Strengths and Limitation

Our TT4 dataset was limited to very preterm infants without severe brain injuries who had one undergone measurement of TT4, showing a serial TSH level < 10.0 μIU/mL. Therefore, these findings might be not generalizable beyond this specific group. In particular, periviable infants (22–25 weeks GA) who undergo longer hospitalization should be separately and specifically focused upon (Supplementary Table S4) [18].

The use of TT4 for screening, as opposed to FT4, may be a limitation of this study. This is because this study was based on the detection of delayed TSH elevation. The use of a single set of TT4 data, instead of multiple TT4 datasets, might be another limitation; however, a serial TSH level < 10.0 μIU/mL might imply a normal thyroid status in those VPIs. Future studies should aim to take multiple measurements of serum FT4 in VPIs at 4–6 weeks postnatal age.

The high follow-up rate (90.4%) of infants at 24 months corrected age was a strength of this study. Furthermore, obtaining the morbidities of preterm infants and the exact PMA provides more information regarding the relationship between TT4 and NDI.

## 5. Conclusions

Thyroid function screening is important for VPIs. However, a single 1-month-old serum TT4 concentration measurement may not be sufficiently clearly associated with long-term neurodevelopmental impairment in neurologically intact VPIs, who have already undergone three normal TSH screenings. The morbidities of prematurity were related to long-term neurodevelopmental impairments. To be able to attribute the NDI of TSH-normal VPIs to a single postnatal TT4 concentration measurement may require more investigation.

## Figures and Tables

**Table 1:** Background clinical characteristics of the study cohort.

*n* for Further Subgroup Analysis	334
Variables	Values
**Maternal and antenatal variables**	
Maternal Age, years	32.2 ± 5.0
Maternal education level (≥college), *n* (%)	192 (57.3)
Antenatal steroid, *n* (%)	292 (87.2)
Antenatal magnesium sulfate, *n* (%)	87 (26.0)
Antenatal antihypertensive drug, *n* (%)	44 (13.2)
Pre-eclampsia, *n* (%)	67 (20)
Gestational diabetes mellitus, *n* (%)	13 (3.9)
Preterm premature rupture of membranes ≥ 18 h, *n* (%)	114 (34.0)
Chorioamnionitis, *n* (%)	14 (4.2)
Placental abruption, *n* (%)	18 (5.4)
**Variables at perinatal period**	
Gestation age, weeks	27.6 ± 1.9
Bodyweight at birth, gram	1025 ± 244
z score of bodyweight at birth	−0.2 ± 0.8
Sex, male, *n* (%)	188 (56.3)
Method of delivery, Cesarean section, *n* (%)	189 (56.6)
Inborn, *n* (%)	285 (85.3)
Multi-pregnancy, *n* (%)	93 (27.5)
Resuscitation at birth (intubation), *n* (%)	29 (8.7)
Apgar score at 1 min	6 (4–7)
Apgar score at 5 min	8 (7–9)
Body temperature at admission, °C	35.8 ± 0.9
First blood sugar, mg/dL	78.3 ± 28.7
pH of first blood gas	7.3 ± 0.1
Early onset sepsis, *n* (%)	8 (2.4)
**Variables during hospital stay**	
Surfactant treated respiratory distress syndrome, *n* (%)	85 (25.4)
Treated hsPDA, *n* (%)	173 (51.6)
Late onset sepsis, *n* (%)	58 (17.4)
Necrotizing enterocolitis ≥ stage 2, *n* (%)	15 (4.5)
Treated retinopathy of prematurity, *n* (%)	27 (8.1)
Chronic lung disease (CLD), *n* (%)	104 (31.1)
Days on invasive ventilation, days	7.1 ± 20.6
Postnatal steroid for CLD, *n* (%)	31 (9.3)
**Variables at thyroid function screen**	
Postnatal age, days	30.3 ± 5.5
Postmenstrual age, week	31.9 ± 1.9
Serum total thyroxine concentration, µg/dL	6.6 ± 1.9
Serum thyroid stimulating hormone, μIU/mL	3.4 ± 1.9
**Variables at discharge**	
Postnatal age, days	70.3 ± 38.9
Post-conception age at discharge, week	37.5 ± 4.6
Z score of bodyweight at discharge	−1.4 ± 1.1
Neurodevelopmental impairment	91 (27.1%)

Values are numbers (%), mean ± standard deviation or median (lower/upper quartiles) if not mentioned. hsPDA: hemodynamic significant patent ductus arteriosus.

**Table 2:** Dependence of neurodevelopmental impairment on clinical variables: univariate analysis.

Variables	Ref.	Odds Ratio	Lower 95% CI	Upper 95% CI	*p*-Value
**Maternal and antenatal variables**					
Maternal age, year		0.993	0.944	1.046	0.801
Maternal school level ≥ college	(Ref. No)	0.433	0.262	0.714	**0.001**
Antenatal steroid	(Ref. No)	0.717	0.324	1.588	0.413
Antenatal magnesium sulfate	(Ref. No)	1.649	0.909	2.992	0.100
Antenatal antihypertensive drug	(Ref. No)	2.626	1.06	6.508	0.037
Pre-eclampsia	(Ref. No)	1.991	1.003	3.955	**0.049**
Gestational diabetes mellitus	(Ref. No)	1.694	0.523	5.486	0.379
Preterm premature rupture of membranes ≥ 18 h	(Ref. No)	1.192	0.792	2.003	0.508
Chorioamnionitis	(Ref. No)	1.167	0.342	3.976	0.805
Placental abruption	(Ref. No)	1.515	0.568	4.042	0.407
**Variables at perinatal period**					
Gestational age; weeks		0.798	0.701	0.908	**0.001**
bodyweight at birth; grams		0.999	0.997	1.000	**0.005**
z score of bodyweight at birth		0.994	0.719	1.375	0.972
Sex, male	(Ref: female)	1.614	0.973	2.676	**0.064**
Method of delivery, CS	(Ref: VD)	0.964	0.586	1.586	0.885
Inborn	(Ref. out-born)	1.141	0.572	0.278	0.708
Multi-pregnancy	(Ref. No)	1.295	0.757	2.214	0.345
Resuscitation at birth (intubation)	(Ref. No)	0.576	0.246	1.349	0.204
Apgar score at 1 min		0.920	0.813	1.042	0.19
Apgar score at 5 min		0.873	0.758	1.005	0.059
Body temperature at birth, °C		0.816	0.611	1.090	0.168
First blood sugar, mg/dL		1.006	0.997	1.016	0.208
pH of first blood gas		0.347	0.020	6.167	0.471
Early onset sepsis	(Ref. No)	1.405	0.328	6.005	0.647
**Variables during hospital stay**					
Surfactant treated respiratory distress syndrome	(Ref. No)	1.945	1.133	3.340	**0.016**
Treated hsPDA	(Ref. No)	2.531	1.513	4.234	**<0.001**
Late onset sepsis	(Ref. No)	0.928	0.481	1.792	0.824
Necrotizing enterocolitis ≥ stage 2	(Ref. No)	4.522	1.471	13.898	**0.008**
Treated retinopathy of prematurity	(Ref. No)	4.288	1.863	9.867	**0.001**
Chronic lung disease (CLD)	(Ref. No)	3.292	1.958	5.532	**<0.001**
Days on invasive ventilation, days		1.055	1.029	1.082	**<0.001**
Postnatal steroid for CLD	(Ref. No)	4.956	2.187	11.232	**<0.001**
**Variables at thyroid function study**					
Serum total thyroxine concentration, µg/dL		0.959	0.845	1.088	0.519
**Variables at discharge**					
Z score of bodyweight at discharge ^1^		0.731	0.589	0.908	**0.005**

CI: confidence interval; No: not diagnosed or treated; Ref: reference; CS: cesarean section; VD: vaginal delivery; ^1^ calculated as postmenstrual age in weeks; hsPDA: hemodynamic significant patent ductus arteriosus. Statistical significance was assumed for *p* < 0.05 (indicated in bold).

**Table 3:** Dependence of neurodevelopmental impairment * on clinical variables: a multivariate analysis.

Covariates	OR	Lower 95% CI	Upper 95% CI	*p*-Value
Gestational age, week	0.939	0.777	1.136	0.519
Sex				
Male	1.889	1.067	3.343	**0.029**
Female (Reference)				
Maternal education level				
level ≥ college	0.407	0.235	0.705	**0.001**
level < college (Reference)				
Apgar score at 5 min	1.000	0.840	1.190	0.999
Cesarean section				
Yes	1.302	0.529	3.206	0.566
No (Reference)				
Surfactant treated respiratory distress syndrome				
Yes	1.079	0.547	2.128	0.826
No (Reference)				
Treated hsPDA				
Yes	1.883	1.002	3.540	**0.049**
No (Reference)				
Postnatal steroid therapy for CLD				
Yes	3.206	1.185	8.671	**0.028**
No (Reference)				
Necrotizing enterocolitis ≥ stage 2				
Yes	3.839	1.142	12.906	**0.030**
No (Reference)				
Treated retinopathy of prematurity				
Yes	2.309	0.861	6.197	0.097
No (Reference)				
Total thyroxine concentration, µg/dL	1.131	0.969	1.320	0.119

OR: odds ratio; CI: confidence interval; hsPDA: hemodynamic significant patent ductus arteriosus. CLD: chronic lung disease. * Neurodevelopmental impairment at 24 months corrected age. Statistical significance was assumed for *p* < 0.05 (indicated in bold).

## Data Availability

The data presented in this study are available on request from the corresponding author. The data are not made publicly available due to patient confidentiality.

## Supplementary Materials

The following are available online at https://www.mdpi.com/2072-6643/13/4/1055/s1, Table S1. The characteristic of 12 very preterm infants treated with L-thyroxin in this study; Table S2. Dependence of serum total thyroxine concentration on clinical variables: multivariate linear regression analysis; Table S3. Dependence of mental performance score on clinical variables: multivariate linear regression analysis; Table S4. Dependence of neurodevelopmental impairment on clinical variables in periviable infants: a multivariate analysis.
