# Supplementary material for: Postnatal Serum Total Thyroxine of Very Preterm Infants and Long-Term Neurodevelopmental Outcome
Source: Nutrients. 2021 Mar 24;13(4):1055. doi: 10.3390/nu13041055 (PMC8064055; doi:10.3390/nu13041055)
Supplement: Supplementary file 1 [file nutrients-13-01055-s001.pdf]

Supplementary Table S1. The characteristics of 12 very preterm infants treated with L-thyroxin in this study

Case	GA	BBW	Stage I	Stage II		Stage III	Survival	Neurodevelopmental Follow-Up at 24 Months Corrected Age							Permanent Congenital Hypothyroidism
			TSH	TSH	TT4	TSH		B-II MDI	B-II PDI	B-III Cog.	B-III Lang.	B-III Motor	CP	NDI	
1	27	610	<10	15.36	5.24	44.4	No [1]	NA	NA	NA	NA	NA	NA	NA	NA
2	23	535	<10	15.7	NA	NA	No [2]	NA	NA	NA	NA	NA	NA	NA	NA
3	25	842	250	958.4	NA	<10	Yes	NA	NA	90	94	91	No	No	No
4	29	1200	45.4	278.3	1.26	<10	Yes	96	80	NA	NA	NA	No	No	No
5	26	850	<10	353.9	0.955	<10	Yes	NA	NA	90	89	88	No	No	No
6	26	730	<10	193.85	0.63	<10	Yes	NA	NA	60	62	49	Yes	Yes [3]	No
7	29	1370	<10	63.86	3.59	<10	Yes	NA	NA	95	94	94	No	No	No
8	27	632	<10	40.71	3.29	<10	Yes	NA	NA	90	86	94	No	No	No
9	25	825	<10	14.12	4.06	<10	Yes	NA	NA	75	86	91	No	Yes [4]	No
10	27	900	<10	9.17	4.35	45.3	Yes	72	80	NA	NA	NA	No	No	No
11	26	530	<10	2.97	1.76	<10	Yes	NA	NA	95	94	91	No	No	No
12	26	1060	<10	1.43	2.54	<10	Yes	NA	NA	85	91	85	No	No	No

GA: gestational age; BBW: birth bodyweight; NA: not available; TT4: serum total thyroxin (μg/dL); TSH: thyroid-stimulation hormone (μIU/L); NDI: neurodevelopmental impairment; MDI: mental developmental index; PDI: physical developmental index; Con: cognition composite score; Lang: language composite score; Mo: motor composite score; CP: cerebral palsy; B-II: Bayley Scales of Infant and Toddler Development, Edition II; B-III: Bayley Scales of Infant and Toddler Development, Edition III; Stage I: first national screen at postnatal day 3-5 ; Stage II: at postnatal 1-month-old; Stage III: second national screen at term equivalent age, a detailed description of the stage presented in the methodology section 2.2.[1] severe chronic lung disease; [2] septic shock; [3] severe birth asphyxia; [4] necrotizing enterocolitis and prolonged hypotension;

Categories of thyroid dysfunction:
1. Primary congenital hypothyroidism: case 3, 4
2. Delayed TSH elevation: case 1, 2, 5–10
3. Suspect transient hypothyroidism of prematurity: 11, 12

Cases detected at:
1. Stage I: case 3, 4
2. Stage II: case 1–2, 5–9, 11–12
3. Stage III: case 10

Supplementary Table S2. Dependence of serum total thyroxine concentration on clinical variables: multivariate linear regression analysis.

Covariates	Mean Coefficient	Lower 95% CI	Upper 95% CI	*p*-Value
Antenatal steroid (Y = 1; N = 0)	0.025	−0.563	0.612	0.934
Antenatal magnesium sulfate (Y = 1; N = 0)	0.216	−0.289	0.72	0.401
Antenatal antihypertensive drug (Y = 1; N = 0)	−0.518	−1.201	0.164	0.136
Pre-eclampsia (Y = 1; N = 0)	−0.883	−1.864	0.097	0.077
Gestational diabetes mellitus (Y = 1; N = 0)	0.25	−0.459	0.958	0.489
Multi-pregnancy (Y = 1; N = 0)	−0.104	−0.550	0.342	0.646
Method of delivery, (CS:1; VD:0)	−0.086	−0.521	0.348	0.696
**Sex, (male 1; female 0)**	−0.721	−1.106	−0.335	**<0.001**
**z score of body weight at birth**	0.528	0.233	0.823	**<0.001**
Apgar score at 5 minutes	0.007	−0.117	0.131	0.911
Surfactant treated RDS (Y = 1; N = 0)	0.179	−0.300	0.659	0.462
Early onset sepsis (Y = 1; N = 0)	−1.001	−2.212	0.211	0.105
Treated hsPDA (Y = 1; N = 0)	−0.191	−0.626	0.245	0.389
Postnatal (<1 month age) steroid (Y = 1; N = 0)	0.478	−0.184	1.139	0.157
**Postmenstrual age of TT4 sampling, week**	0.459	0.331	0.587	**<0.001**

Y: treated or diagnosed; N: non-treated or non-diagnosed; CS: cesarean section; VD: vaginal delivery; hsPDA: hemodynamic significant patent ductus arteriosus; RDS: respiratory distress syndrome. Statistical significance was assumed for $p < 0.05$ (indicated in bold).

Supplementary Table S3. Dependence of mental performance score* on clinical variables: multivariate linear regression analysis.

Covariates	Mean Coefficient	Lower 95% CI	Upper 95% CI	*p*-Value
Gestational age, week	0.861	−0.246	1.967	0.127
Sex (male = 1; female = 0)	−4.970	−8.175	−1.765	**0.002**
**Maternal education level**<br>level ≥ college = 1<br>level < college = 0	8.513	5.337	11.689	<0.001
Apgar score at 5 minutes	−0.160	−1.191	0.87	0.76
Method of delivery, (CS:1; VD:0)	−2.485	−5.783	0.812	0.139
Surfactant treated RDS (Y = 1; N = 0)	0.559	−3.512	4.629	0.787
Treated hsPDA (Y = 1; N = 0)	−0.944	−4.568	2.68	0.609
Postnatal steroid therapy for CLD (Y = 1; N = 0)	−5.878	−12.114	0.359	0.065
**Necrotizing enterocolitis ≥ stage 2 (Y = 1; N = 0)**	−7.984	−15.636	−0.332	**0.041**
Treated retinopathy of prematurity (Y = 1; N = 0)	−4.482	−10.899	1.935	0.17
Total thyroxine concentration, µg/dL	−0.407	−1.273	0.459	0.356

Y: treated or diagnosed; N: non-treated or non-diagnosed; CS: cesarean section; VD: vaginal delivery; hsPDA: hemodynamic significant patent ductus arteriosus; RDS: respiratory distress syndrome. CLD: chronic lung disease. * mental performance score described in section 2.4 of Materials and Methods; Statistical significance was assumed for $p < 0.05$ (indicated in bold).

Supplementary Table S4. Dependence of neurodevelopmental impairment* on clinical variables in periviable infants: a multivariate analysis.

Study Population	All infants in the cohort (n = 334)				GA ≤ 25 weeks (n = 56 [a])			
**Covariates**	**OR**	**Lower 95% CI**	**Upper 95% CI**	***p*-Value**	**OR**	**Lower 95% CI**	**Upper 95% CI**	***p*-Value**
Gestational age, week	0.882	0.697	1.116	0.295				
Gestational group<br>≤25 weeks<br>> 25 weeks (Reference)	0.612	0.209	1.79	0.37				
Sex<br>Male<br>Female (Reference)	1.869	1.055	3.311	**0.032**				
Maternal education level<br>level ≥ college<br>level < college (Reference)	0.4	0.23	0.694	**0.001**	0.756	0.235	2.434	0.639
Apgar score at 5 minutes	1.001	0.841	1.191	0.992				
Cesarean section<br>Yes<br>No (Reference)	1.206	0.678	2.145	0.525				
Surfactant treated respiratory distress syndrome<br>Yes<br>No (Reference)	1.074	0.544	2.12	1.074				
Treated hsPDA<br>Yes<br>No (Reference)	1.889	1.005	3.549	**0.048**				
Postnatal steroid therapy for CLD<br>Yes<br>No (Reference)	3.485	1.269	9.572	**0.015**	3.74	1.116	12.531	**0.033**
Necrotizing enterocolitis ≥ stage 2<br>Yes<br>No (Reference)	3.869	1.155	12.967	**0.028**				
Treated retinopathy of prematurity<br>Yes<br>No (Reference)	2.464	0.902	6.733	0.079				
Total thyroxine concentration, μg/dL	1.124	0.963	1.313	0.139	0.769	0.517	1.143	0.194

* neurodevelopmental impairment at 24 months corrected age; OR: odds ratio; CI: confidence interval; hsPDA: hemodynamic significant patent ductus arteriosus; CLD: chronic lung disease; [a] 24 infants with neurodevelopmental impairment; Statistical significance was assumed for $p < 0.05$ (indicated in bold).
